# Bim Expression Promotes the Clearance of Mononuclear Phagocytes during Choroidal Neovascularization, Mitigating Scar Formation in Mice

**DOI:** 10.3390/life12020208

**Published:** 2022-01-29

**Authors:** Shoujian Wang, Ismail S. Zaitoun, Soesiawati R. Darjatmoko, Nader Sheibani, Christine M. Sorenson

**Affiliations:** 1 Department of Ophthalmology and Visual Sciences, University of Wisconsin School of Medicine and Public Health, Madison, WI 53705, USA; shoujianwang@wisc.edu (S.W.); iszaitoun@wisc.edu (I.S.Z.); srdarjat@wisc.edu (S.R.D.); nsheibanikar@wisc.edu (N.S.); 2 McPherson Eye Research Institute, University of Wisconsin School of Medicine and Public Health, Madison, WI 53705, USA; 3 Department of Pediatrics, University of Wisconsin School of Medicine and Public Health, Madison, WI 53705, USA

**Keywords:** angiogenesis, vascular dysfunction, inflammation, macrophages, age-related macular degeneration, choroid, Bcl-2 family

## Abstract

Inflammation is increasingly recognized as an important modulator in the pathogenesis of neovascular age-related macular degeneration (nAMD). Although significant progress has been made in delineating the pathways that contribute to the recruitment of inflammatory cells and their contribution to nAMD, we know little about what drives the resolution of these inflammatory responses. Gaining a better understanding of how immune cells are cleared in the choroid will give a novel insight into how sustained inflammation could influence the pathogenesis of nAMD. The pro-apoptotic Bcl-2 family member Bim is a master regulator of immune cell homeostasis. In its absence, immune cell lifespan and numbers increase. Most therapeutic regimes that squelch inflammation do so by enhancing immune cell apoptosis through enhanced Bim expression and activity. To test the hypothesis that Bim expression tempers inflammation during the pathogenesis of nAMD, we used the mouse laser-induced choroidal neovascularization (CNV) model in which inflammation acts as a facilitator of CNV. Here, we showed minimal to no change in the recruitment of F4/80-, CD80-, CD11b-, and Iba1-positive myeloid-derived mononuclear phagocytes to the site of laser photocoagulation in the absence of Bim expression. However, the resolution of these cells from the choroid of Bim-deficient (Bim -/-) mice was significantly diminished following laser photocoagulation. With time, we noted increased scar formation, demonstrated by collagen I staining, in Bim -/- mice with no change in the resolution of neovascularization compared to wild-type littermates. We also noted that mice lacking Bim expression in mononuclear phagocytes (Bim^Flox/Flox^; Lyz2-Cre (Bim^MP^) mice) had delayed resolution of F4/80-, CD80-, CD11b-, and Iba1-positive cells, while those lacking Bim expression in endothelial cells (Bim^Flox/Flox^; Cad5-Cre (Bim^EC^) mice) had delayed resolution of only CD11b- and Iba1-positive cells. Both Bim^MP^ and Bim^EC^ mice demonstrated increased scar formation, albeit to differing degrees. Thus, our studies show that resolving inflammation plays an important role in moderating scar formation in nAMD, and it is impacted by Bim expression in both the endothelium and mononuclear phagocyte lineages.

## 1. Introduction

Inflammation plays a central role in the pathogenesis of neovascular eye diseases including exudative/neovascular age-related macular degeneration (nAMD), retinopathy of prematurity (ROP), and diabetic retinopathy. AMD is the major cause of vision loss among aging Americans. The visual deficit that initially arises from retinal degeneration (dry AMD) is often complicated by the secondary effects of choroidal neovascularization (CNV) (wet or nAMD) [1]. The choriocapillaris, which lies beneath the retinal pigment epithelium (RPE), has the highest flow rate in the body [2] and plays an important role in vision health. Maintenance of the choroidal circulation facilitates photoreceptor and outer retinal health, and its disruption contributes to the pathogenesis of sight-threatening eye diseases such as AMD. CNV-related neovascularization, originating in the choroid, extends through the Bruch’s membrane to the RPE layer, with the loss of the RPE and photoreceptor cells compromising vision [3]. Although vascular endothelial growth factor (VEGF) expression is essential for the maintenance of the RPE and the health of the choriocapillaris, its increased expression contributes to CNV and leakiness of the choroidal vessels. RPE atrophy is noted in about 20–25% of patients receiving anti-VEGF treatment, further demonstrating the essential role VEGF plays in tissue development and homeostasis [4,5]. Thus, maintaining choroidal vascular homoeostasis is essential for normal vision.

Mononuclear phagocytes (MPs) support innate and adaptive immune responses and have an important role in the maintenance of immune homeostasis. MPs such as macrophages can have a detrimental impact in eye diseases, including nAMD and experimental autoimmune uveoretinitis [6]. In AMD, MP accumulation in the subretinal space influences neovascularization and ultimately RPE and photoreceptor degeneration [7,8]. Macrophage recruitment has also been shown to play an important role in CNV. Mice lacking monocyte chemoattractant protein 1/CCL2 receptor (*Ccr*2-deficient mice) demonstrated a significant decrease in early macrophage recruitment and subsequent CNV compared to control mice [9]. In contrast, thrombospondin-1-deficient (*Thbs1*-/-) mice demonstrated a significant increase in the recruitment of F4/80-positive macrophages three days after laser photocoagulation and subsequently increased CNV [10]. Thus, the initial recruitment of MPs can influence disease pathogenesis, and their persistence may further exaggerate these responses. 

Bim is a proapoptotic Bcl-2 family member that plays an important role in the remodeling of the developing retinal vasculature and pathological vessel loss observed during hyperoxia [11,12]. The removal of unnecessary cells or those with imperfections or damage is essential for tissue homeostasis. Bim is touted as a master regulator for immune cell homeostasis. A lack of Bim expression increases myeloid and lymphoid cell numbers and disrupts thymocyte development [13,14]. Bim expression modulates inflammation, influencing the clearance of macrophage and mast cells [15]. Mice globally lacking Bim expression demonstrate autoimmune disease, with Bim-deficient macrophages having increased markers of macrophage activation and elevated cytokine levels [16]. However, the role that modulators of apoptosis such as Bim play in the development, maintenance, and modulation of inflammation in diseases of the choroid has not been delineated.

Here, we examined the influence of Bim expression for MP recruitment and clearance in the choroid. We utilized the laser induced CNV model, in which inflammation facilitates CNV, to assess whether Bim expression tempers the inflammatory responses during CNV. We showed a similar recruitment of F4/80-, CD80-, and CD11b-positive MPs to the site of laser photocoagulation in wild-type and Bim -/- littermates. We did note a modest increase in the recruitment of Iba1-positive cells in the absence of Bim. The resolution of these immune cells was, however, delayed in the absence of Bim and was concomitant with increased scar formation over time. The CNV level was not affected. We also showed that a lack of Bim expression in myeloid-derived MPs was sufficient to diminish the resolution of F4/80-, CD80-, Iba1-, and CD11b-positive cells and increase scar formation. Thus, in the absence of Bim expression, slackened MP resolution following laser photocoagulation correlates with increased scar formation but not neovascularization. 

## 2. Results

### 2.1. Bim Modulates Resolution of F4/80-Positive Staining Cells

The pathogenesis of nAMD results from a futile circle of inflammation, neovascularization, and scarring that leads to vision loss. Patient variability associated with disease progression is not well understood. Although the correlation between inflammation and nAMD is well documented, our understanding of the connection between these processes is yet emerging. 

**Figure 1:**
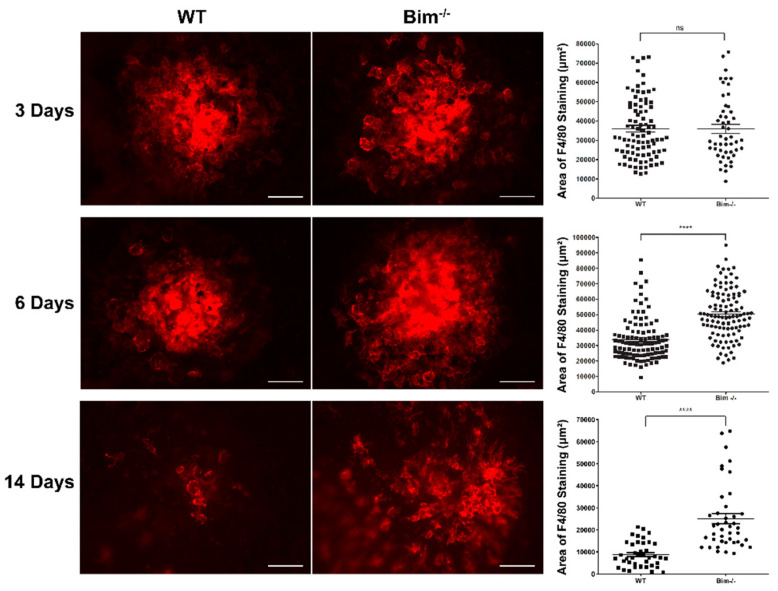
Decreased resolution of F4/80 cells in the absence of Bim expression. Choroidal neovascularization was induced in 3-month-old wild-type (WT) and Bim -/- mice by laser-photocoagulation-induced rupture of Bruch’s membrane. At 3, 6, and 14 days following laser photocoagulation, the eyes were sectioned at the equator and the anterior half with the vitreous and retina discarded. The remaining RPE/choroid complex was stained with anti-F4/80, and the positive staining area quantified (ns; not significant; **** *p* < 0.0001). Each point represents one laser spot. Scale bar = 100 µm. These experiments were repeated with at least 8 mice of each genotype.

To maintain tissue integrity, the resolution of inflammation is key. Since local apoptosis plays a fundamental role in the removal of macrophages in other locales [17], here we addressed the role that Bim expression plays in the choroid for the removal and/or clearance of MPs following laser-induced photocoagulation. Wild-type and Bim -/- littermates underwent laser-induced rupture of the Bruch’s membrane by photocoagulation. To assess MP recruitment and resolution, F4/80 staining of the RPE-choroid complex was performed 3, 6, and 14 days following laser photocoagulation, and the F4/80-positive cells were quantitated (Figure 1). Typically, 3 days following laser photocoagulation shows the peak MP recruitment. At 3 days post-laser the level of F4/80-positive staining was similar in wild-type and Bim -/- littermates. However, at 6 days post-laser the level of F4/80-positive staining increased in Bim -/- mice, while in wild-type littermates F4/80 staining remained similar to day 3 (WT 33,887 ± 1350 vs. Bim -/- 50,422 ± 1506; **** *p* < 0.0001). At 14 days following laser photocoagulation there was a precipitous drop in the level of F4/80 staining in the choroid of wild-type mice (76% decline from 3 days; WT 8746 ± 892), signaling the major resolution of these cells, while the decline in Bim -/- mice at 14 days was significantly less (Bim -/- 25,088 ± 2311) (Figure 1). Thus, not only did F4/80-positive cells not adequately resolve from the choroid in Bim -/- mice following laser photocoagulation, they actually accumulated to higher levels after 6 days.

### 2.2. Bim Expression Aids Resolution of CD11b-Positive Staining Cells

CD11b is the ligand binding subunit for the dimeric integrin CD11b/CD18 (integrin α_M_β_2_) and the receptor for ICAM-1 and fibrinogen. It plays a key role in the phagocytosis of apoptotic cells by limiting inflammatory responses and negatively regulating proinflammatory pathways in tissue, including the Toll-like receptor (TLR) and Fc receptor γ [18,19,20,21,22]. CD11b not only mediates phagocytosis and survival but also cell adhesion, migration, and chemotaxis [23,24,25]. Thus, CD11b is essential for homeostasis and tolerance as well as the downregulation of inflammation.

**Figure 2:**
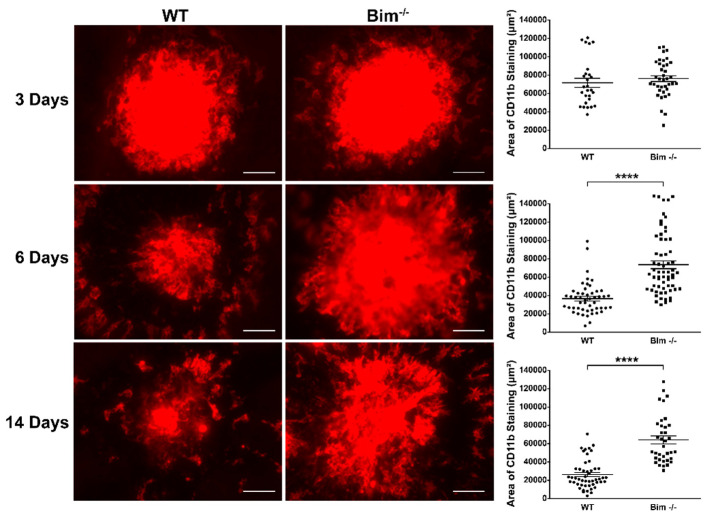
Increased CD11b-positive cells at 6 days following laser photocoagulation in Bim -/- mice. RPE/choroid complex was harvested from 3-month-old wild-type and Bim -/- mice 3, 6, and 14 days following laser photocoagulation. The RPE/choroid complex was wholemount stained with anti-CD11b and the positive staining area quantified (**** *p* < 0.0001). Each point represents one laser spot. Scale bar = 100 µm. These experiments were repeated with at least 8 mice of each genotype.

**Figure 3:**
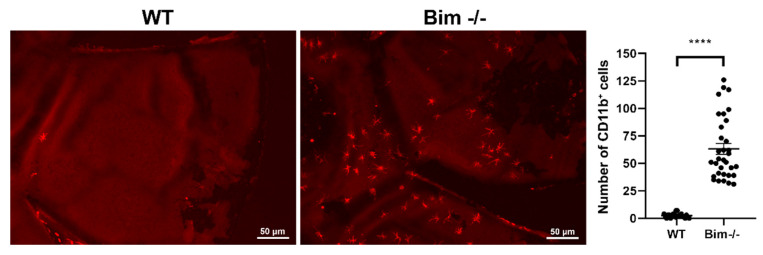
Increased number of CD11b-positive cells in Bim -/- naïve choroid. RPE/choroid complex was harvested from naïve 8-week-old wild-type (WT) and Bim -/- mice. The complex was wholemount stained with anti-CD11b and the positive staining area quantified. Each point represents one RPE/choroid complex. These experiments were repeated with at least 6 mice of each genotype. Scale bar = 50 µm. (**** *p* < 0.0001).

To assess the state of macrophages following laser photocoagulation, here we assessed wholemount CD11b staining (M2, anti-inflammatory macrophages) in the RPE–choroid complex from wild-type and Bim -/- littermates following laser photocoagulation (Figure 2). At 3 days following laser photocoagulation no significant difference was observed between CD11b levels in wild-type and Bim -/- mice (WT 71,554 ± 4947 vs. Bim -/- 76,189 ± 323). In wild-type mice, CD11b levels decreased at 6 and 14 days following laser photocoagulation (36,390 ± 2455 (6 days) vs. 26,323 ± 2238 (14 days)). In contrast, minimal if any declines in CD11b levels were observed in the choroid from Bim -/- mice that underwent laser photocoagulation (76,189 ± 323 (3 day), 73,356 ± 4184 (6 days) vs. 64,177 ± 4332 (14 days)). These data demonstrate a fundamental role for Bim expression in the resolution of CD11b-positive cells. Since we noted CD11b-positive cells in the choroid from Bim -/- mice peripheral to the area of photocoagulation, we next asked whether naïve Bim -/- mice RPE/choroid preparations demonstrated increased CD11b-positive cells. Figure 3 demonstrates a significant increase in the numbers of CD11b-positive cells in the choroid from naïve Bim -/- mice compared to wild-type mice. Thus, significant numbers of CD11b-positive cells are present in the choroid of naïve Bim -/- mice, and their accessibility may aid their sustained presence in the absence of Bim in mice subjected to laser photocoagulation.

### 2.3. Delayed Clearing of CD80-Positive Staining Cells in the Absence of Bim

**Figure 4:**
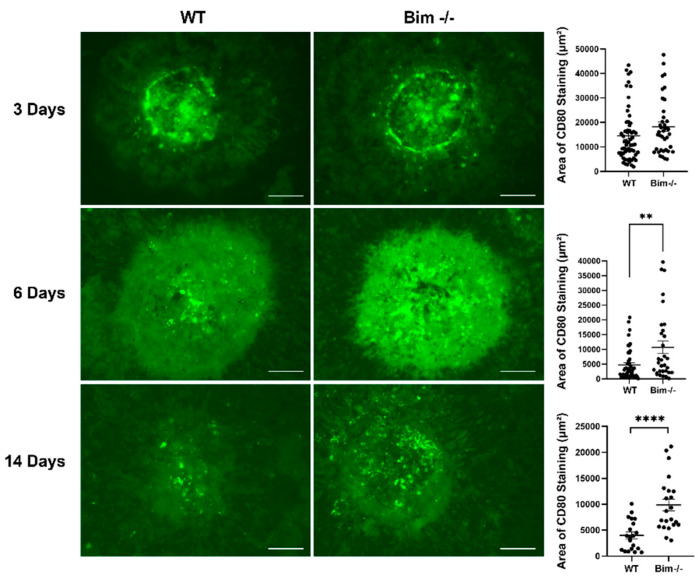
Decreased resolution of CD80-positive cells in RPE/choroid from Bim -/- mice. Three-month-old wild-type and Bim -/- mice underwent laser photocoagulation. The choroid RPE/choroid complex was harvested after 3, 6, and 14 days, wholemount stained with anti-CD80, and the positive staining area was quantified. Each point represents one laser spot. These experiments were repeated with at least 8 mice of each genotype. Scale bar = 100 µm. (** *p* < 0.01, **** *p* < 0.0001).

M1 macrophages are inflammatory macrophages that, once activated, produce proinflammatory cytokines and nitric oxide [26]. Although M1 macrophages aid debris removal by their phagocytic and proteolytic properties, their sustained presence leads to prolonged inflammation and tissue degeneration. Here, we assessed CD80 staining in the RPE/choroid complex of wild-type and Bim -/- mice subjected to laser photocoagulation. The amount of CD80 staining cells recruited in the RPE/choroid complex at 3 days following laser photocoagulation was not significantly different in wild-type and Bim -/- mice (WT 14,516 ± 1405 vs. Bim -/- 18,241 ± 1853 (Figure 4)). In contrast to wild-type mice, which demonstrated the resolution of CD80-positive cells, there was a significant delay in the absence of Bim (WT 4001 ± 656 vs. Bim -/- 9857 ± 1130 (14 days); **** *p* < 0.0001). These data illustrate the important role of Bim expression in the resolution of CD80-positive cells following laser photocoagulation.

### 2.4. Delayed Resolution of Iba1-Positive Cells in the Absence of Bim

**Figure 5:**
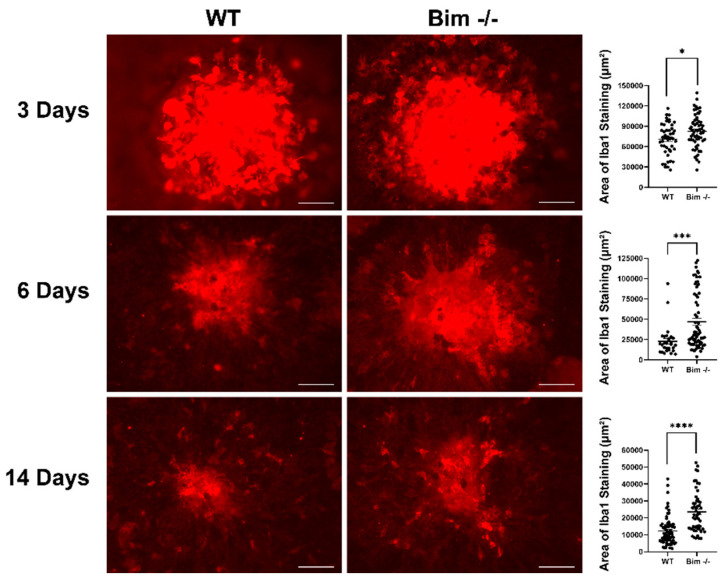
Global lack of Bim expression increased Iba1-positive cells. Wild-type and Bim -/- mice (3-month-old) underwent laser photocoagulation, and the RPE/choroid was harvested 3, 6, and 14 days later. The RPE/choroid was wholemount stained with anti-Iba1 and the positive staining area was quantified. Each point represents one laser spot. These experiments were repeated with at least 8 mice from each genotype. Scale bar = 100 µm. (* *p* < 0.05, *** *p* < 0.001, **** *p* < 0.0001).

**Figure 6:**
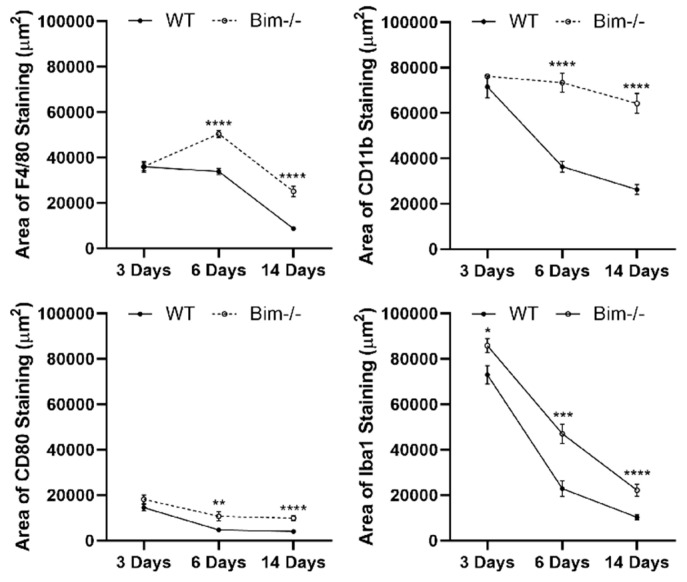
Mice lacking Bim expression have similar recruitment but prolonged resolution of MPs compared to WT counterparts. To further illustrate MP recruitment and clearance with time, the data in Figure 1, Figure 2, Figure 4 and Figure 5 were compiled into line graphs. ** *p* < 0.01, *** *p* < 0.001, **** *p* < 0.0001.

Microglia and choroidal macrophages are activated in response to danger signals and invade by migrating toward the signal. Should activation be sustained, the release of angiogenic and inflammatory factors ensues, leading to neuronal and RPE degeneration [27,28]. Here, we assessed Iba1-positive staining cells following laser photocoagulation in RPE/choroid preparations from wild-type and Bim -/- mice. At 3 days following laser photocoagulation, Bim -/- mice had a modest yet significant increase in the Iba1-positive staining cells in the choroid (WT 73,069 ± 4028 vs. Bim -/- 85,848 ± 3199; * *p* < 0.05 (Figure 5)). In wild-type mice, the number of positive staining cells decreased more rapidly, showing nearly twice the percentage decline in Iba1-positive staining cells compared to Bim -/- mice during the 14 days following laser photocoagulation (WT 10,263 ± 1146 vs. Bim -/- 22,229 ± 2718; **** *p* < 0.0001(Figure 5)). Here, we showed that Iba1-positive staining cells not only have a modest increased recruitment in the absence of Bim, but are also slower to resolve than in their wild-type counterparts. To better illustrate the time-dependent recruitment and clearance of MPs (Figure 1, Figure 2, Figure 4 and Figure 5), the data are summarized in Figure 6.

### 2.5. Lack of Bim Expression in Endothelial Cells or Mononuclear Phagocytes 

**Figure 7:**
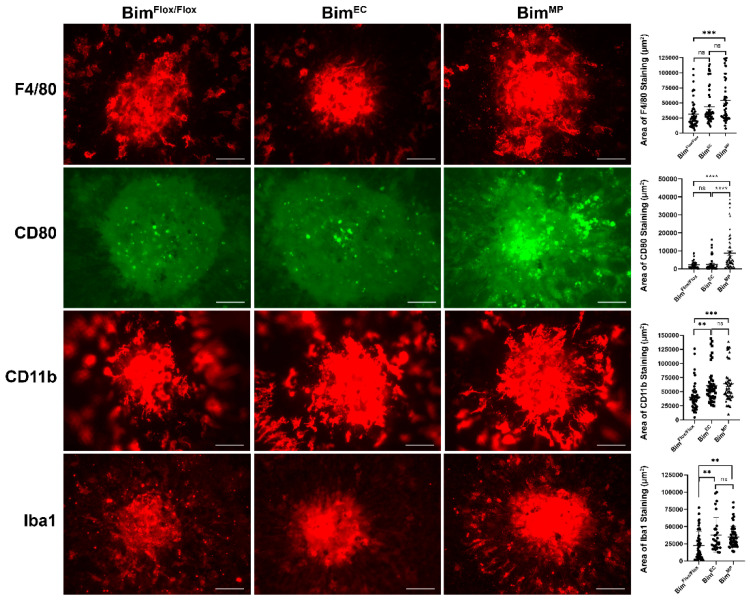
Mice conditionally lacking Bim expression in MPs have decreased resolution of F4/80-, CD80-, CD11b-, and Iba1-positive cells. To assess the role Bim expression plays in moderating the immune response during CNV, 3-month-old Bim^Flox/Flox^, Bim^EC^, and Bim^MP^ mice underwent laser photocoagulation. Six days later, the RPE/choroid was wholemount stained with anti-F4/80, anti-CD80, anti-CD11b, or anti-Iba1, and the area of positive staining was quantified (on the right). Each point represents one laser spot. Experiments were performed with at least 8 mice for each condition. Scale bar = 100 µm. (ns; not significant; **** *p* < 0.0001, *** *p* < 0.001, ** *p* < 0.01).

Choroidal neovascularization relies on contributions from both endothelial cells and MPs. The contribution of Bim expression in these cell types to the inflammatory status of the choroid is not well understood. Our results in mice globally lacking Bim showed little if any difference in myeloid-derived MP recruitment to the site of choroid injury. In contrast, the resolution of these inflammatory cells was significantly diminished in the absence of Bim (Figure 1, Figure 2, Figure 4 and Figure 5). Next, we investigated the contribution that Bim expression in endothelial cells or MPs provided for the resolution of these inflammatory cells. We chose here to examine MP levels 6 days post-laser photocoagulation, when we typically saw decreasing numbers of inflammatory cells in wild-type mice but sustained numbers in Bim -/- mice. The RPE/choroid from Bim^Flox/Flox^ and Bim^EC^ (lacking Bim expression in endothelial cells) mice that had undergone laser photocoagulation 6 days earlier did not demonstrate a significant difference in F4/80 staining levels (Bim^Flox/Flox^ 31,177 ± 3300 vs. Bim^EC^ 43,523 ± 4196). In comparison, RPE/choroid from Bim^MP^ mice (lacking Bim expression in MP) 6 days following laser photocoagulation demonstrated a significant increase in F4/80-positive staining compared to the Bim^Flox/Flox^ littermates (Bim^Flox/Flox^ 31,177 ± 3300 vs. Bim^MP^ 54,299 ± 4915; *** *p* < 0.001 (Figure 7)). Similarly, the CD80 staining of RPE/choroid from these mice only demonstrated increased numbers of CD80-positive staining cells 6 days following laser photocoagulation in Bim^MP^ mice compared to their control littermates or Bim^EC^ mice (Bim^Flox/Flox^ 2286 ± 328 vs. Bim^EC^ 2540 ± 550 vs. Bim^MP^ 8732 ± 1254; **** *p* < 0.0001 (Figure 7)). However, when we assessed CD11b- (Figure 7) and Iba1-positive (Figure 7) staining cells 6 days following laser photocoagulation we noted that the lack of Bim expression in either endothelial cells or MPs delayed the resolution compared to Bim^Flox/Flox^ littermates (CD11b; Bim^Flox/Flox^ 40,318 ± 3353 vs. Bim^EC^ 60,804 ± 4445 vs. Bim^MP^ 64,240 ± 4870; ** *p* < 0.01, *** *p* < 0.001). Thus, a lack of Bim expression in MPs was sufficient to restrain the resolution of F4/80-, CD11b-, Iba1-, and CD80-positive cells following laser photocoagulation.

### 2.6. Increased Scar Formation in the Global Absence of Bim

**Figure 8:**
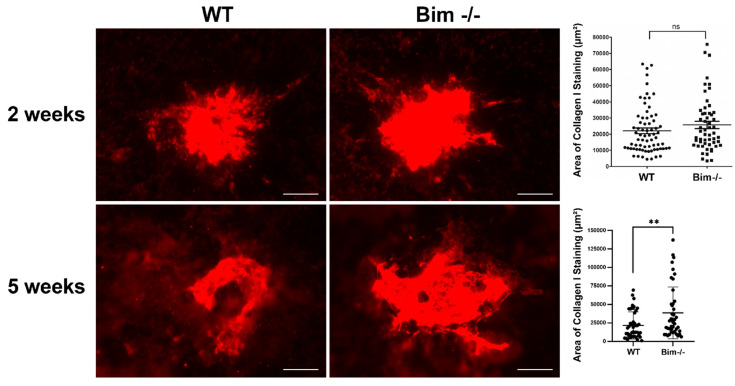
Increased collagen I in the absence of Bim. To assess the amount of scarring, the RPE/choroid from wild-type and Bim -/- mice was wholemount stained with anti-collagen I, 2 and 5 weeks following laser photocoagulation. The area of collagen staining was quantified (on the right). Each point represents one laser spot. At least 8 mice for each condition were used. Scale bar = 100 µm. (ns; not significant; ** *p* < 0.01).

**Figure 9:**
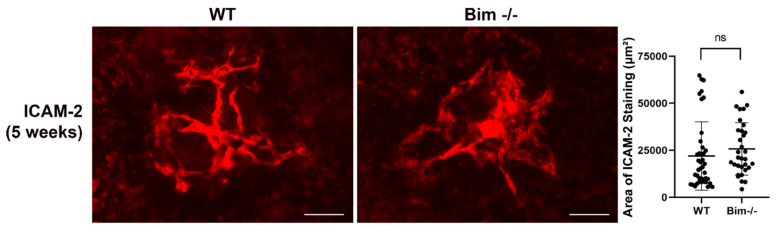
Similar level of CNV was noted 5 weeks following laser photocoagulation in and wild type and Bim -/- mice. Wild-type and Bim -/- mice (3-month-old) underwent laser photocoagulation. Five weeks later, the RPE/choroid was harvested, wholemount stained with anti-ICAM-2, and the area of staining quantified. Each point represents one laser spot. At least 8 mice for each condition were used. Scale bar = 100 µm. (ns; not significant).

Inflammation, particularly sustained inflammation, is considered to contribute to scar formation and subsequent vision loss. Here, we assessed the level of collagen I staining as a measure of scar formation 2 and 5 weeks following laser photocoagulation (Figure 8). These time points were chosen to assess scar formation when CNV had reached its reported maximum (2 weeks) and later to assess ongoing scar formation (5 weeks). We did not observe a significant difference in the collagen I level in RPE/choroid preparations from wild-type and Bim -/- mice 2 weeks following laser photocoagulation. However, at 5 weeks following laser photocoagulation, a significant increase in collagen I staining was noted in the choroid of Bim -/- mice compared to their wild-type littermates. Next, we assessed the level of CNV at 5 weeks following laser photocoagulation. The level of CNV in wild-type and Bim -/- mice was similar at 5 weeks following laser photocoagulation (Figure 9). We had previously shown no significant change in the level of CNV at 2 weeks following laser photocoagulation in wild-type and Bim -/- littermates [11]. Thus, our data indicate continued scar accumulation in the absence of Bim expression without a significant difference in the level of CNV. 

### 2.7. Lack of Endothelial Cell or MP Bim Expression Influences Scar Accumulation

**Figure 10:**
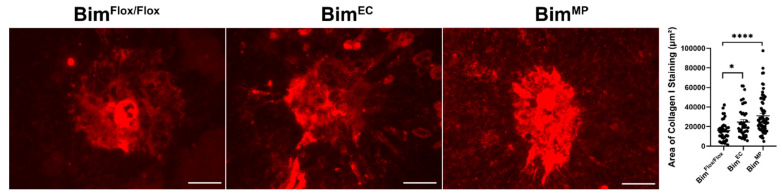
Increased scar accumulation in mice conditionally lacking Bim. Three-month-old Bim^Flox/Flox^, Bim^EC^, and Bim^MP^ mice were subjected to laser photocoagulation. Five weeks following laser photocoagulation, the RPE/choroid was harvested, stained with anti-collagen I, and the area of staining was quantified. Each point represents one laser spot. For each condition at least 8 mice were used. Scale bar = 100 µm. (* *p* < 0.05, **** *p* < 0.0001).

Here, our studies demonstrated that a global lack of Bim expression diminished MP resolution and enhanced ongoing scar formation (5 weeks post laser photocoagulation), without a significant impact on CNV. While Bim^MP^ mice had reduced MP resolution for all markers examined, Bim^EC^ mice had reduced resolution of only CD11b- and Iba1-positive staining cells. To assess scar formation, Bim^Flox/Flox^, Bim^EC^, and Bim^MP^ mice underwent laser photocoagulation followed by wholemount staining of the choroid/RPE with anti-collagen I after 5 weeks. Figure 10 demonstrates enhanced scar accumulation in both Bim^EC^ and Bim^MP^ mice 5 weeks following laser photocoagulation, with Bim^MP^ mice demonstrating the highest amount (Bim^Flox/Flox^ 15,991 ± 1615 vs. Bim^EC^ 24,657 ± 2409 vs. Bim^MP^ 31,074 ± 2327; * *p* < 0.05, **** *p* < 0.0001). Thus, the inability to clear MPs in the absence of Bim is associated with the enhanced severity of scar accumulation. 

## 3. Discussion

The immune system maintains tissue homeostasis by deflecting tissue insults. Inflammation that results from an insult to the tissue acts in a protective capacity for that tissue. Unfortunately, disrupting this protective mechanism is detrimental to tissue integrity and function. Although it is well accepted that inflammation plays a causative role in the pathogenesis of nAMD, the key regulatory events involved in this context require more investigation. Delineating the mechanisms involved, in not only macrophage recruitment, but also their activation and resolution during nAMD, will yield gains in this area. 

Bim is touted as the master regulator of immune cell homeostasis [13,14]. The development and maintenance of the immune system requires the proper function of the intrinsic apoptotic pathway. The imbalance of this pathway can contribute to immunodeficiency, lymphoproliferation, or autoimmune diseases such as systemic lupus erythematosus (SLE) [13]. The propensity of Bim -/- mice to develop autoimmune disease is the product of their elevated lymphoid and myeloid cell numbers as well as disrupted T-cell development. Mice lacking Bim expression in myeloid-derived MPs develop SLE [16], further demonstrating the important role Bim plays in leukocyte homeostasis. Although Bim makes a major contribution to the death of macrophages by apoptosis, Bim also has nonapoptotic functions in macrophages and dendritic cells [29,30,31,32,33,34,35,36]. Therefore, even though the contribution of Bim expression in immune cells is well documented, little is known regarding its role in modulating inflammation associated with the pathogenesis of nAMD. 

Myeloid-derived MPs not only influence inflammation but are also proposed to have an influential role in stimulating angiogenesis during nAMD. However, there are many facets to the overall umbrella of this inflammatory process to consider, including the recruitment of MPs, the numbers of M1 and M2 inflammatory cells, and the resolution of these MPs, to name a few. The path from inflammation to neovascularization and subsequent scarring with vision impairment may not be straightforward when these multiple facets are considered. Several studies have linked the enhanced recruitment of MPs to enhanced CNV in the laser photocoagulation mouse model. Mice lacking TSP1, an endogenous inhibitor of angiogenesis and inflammation, demonstrated increased macrophage recruitment, which correlated with increased CNV following laser rupture of the Bruch’s membrane [10]. We recently showed that this effect of global TSP1 deficiency is mimicked by the lack of TSP1 in endothelial cells and MPs [37]. In contrast, a lack of CCR2 causes a decline in the number of infiltrating macrophages with a decreased area of CNV [9]. We showed a similar recruitment of MPs in both wild-type and Bim -/- mice following laser photocoagulation and similar levels of CNV in these mice [11]. Thus, MP recruitment correlates well with CNV levels.

In order to maintain tissue integrity, the ability to resolve inflammation is key. We sought here to address whether Bim expression was critical for immune cell resolution and its contribution to CNV pathogenesis. Although the recruitment of MPs (F4/80-, CD80-, and CD11b-positive cells) was similar following laser photocoagulation of wild-type and Bim -/- mice, the resolution of these immune cells was greatly diminished in the absence of Bim. These data further exemplify Bim’s role as a master regulator of immune cell homeostasis. When we assessed the role that Bim expression in endothelial cells or MPs plays during CNV, we noted that mice lacking Bim expression in endothelial cells demonstrated a delayed resolution of F4/80- and CD80-positive cells, while mice lacking Bim expression in MPs showed a delayed resolution of all immune cells examined here. The impact that the loss of Bim expression in endothelial cells has in modulating the resolution of CD11b- or Iba1-positive cells may implicate Bim in regulating endothelial cell inflammatory responses, such as the production of MCP1 and ICAM-1 expression. This would allow the recruitment of CCR2^+^ monocytes to the site of inflammation. Thus, our data indicate that Bim expression is essential for the clearance of MPs, thus preventing sustained inflammation. 

The importance of the regulation of immune cell resolution and its implications for disease have recently become a matter of greater interest. For example, CFH Y402H variant expression in MPs interferes with the effective interaction of TSP1 with its receptor CD47 to enhance their clearance, exacerbating inflammation [8,10]. Here, we showed that one ramification of sustained inflammation is continued scar accumulation following laser photocoagulation, as assessed by collagen I staining. Unlike neovascularization, which reaches its peak at 1–2 weeks following laser rupture of the Bruch’s membrane, the associated scar continues to form, leading to traction and potential loss of vision. Here, we noted that the delayed resolution of immune cells in the absence of Bim expression correlated with increased choroidal scarring over time (5 weeks). The levels of collagen I staining in wild-type and Bim -/- mice 2 weeks following laser photocoagulation were similar. We previously showed similar levels of CNV at 2 weeks following laser photocoagulation [11], and here this was demonstrated at 5 weeks in wild-type and Bim -/- mice. We also noted that in Bim^EC^ and Bim^MP^ mice, the delayed clearance of MPs correlated with scar accumulation. Therefore, the studies presented here correlate choroidal scarring with the clearance of immune cells by Bim but not with the level of CNV. 

Bim expression is necessary for many therapeutic regimens that remove unwanted cells via apoptosis. Responsiveness to drugs such as anti-VEGF, corticosteroids, and tyrosine kinase inhibitors hinge on Bim function, with Bim polymorphisms negating effective treatment. Individuals carrying a C→T substitution (c456C > T; T allele BIM BH3 single nucleotide polymorphism (SNP)) within the BIM BH3 domain (death-initiating domain) have lower basal Bim expression [38], while those with the C allele have higher basal Bim expression. Patients with a C allele in the BIM BH3 SNP have a greater sensitivity to corticosteroid treatment and an increased incidence of osteonecrosis [39]. This synonymous Bim polymorphism has a C allele frequency that varies from 43 to 87%, depending on the population [38,40,41]. Though less common, a Bim-deletion polymorphism (BIM BH3 domain spliced out) has been identified in individuals of East Asian (12.3%) and Hispanic (15.7%) descent [38]. Individuals carrying this deletion polymorphism do not produce a functional Bim protein, while individuals with the BIM BH3 SNP T allele have decreased Bim expression. Here, we showed that the lack of Bim expression in mice correlates with sustained inflammation and scarring during CNV. Thus, our results suggest that patients with these BIM SNPs that have decreased Bim expression will have an increased potential for vision loss with nAMD due to sustained inflammation and increased scarring. This may also explain the lack of response to anti-VEGF noted in 30% of patients with nAMD. 

In summary, the studies presented here demonstrate the importance of resolving inflammation to prevent continuing scar formation. We show that Bim expression in both MPs and endothelial cells plays an essential role in immune cell resolution, preventing increased scar formation. In this context, Bim expression appears to act as a senolytic, relieving inflammation and scarring. Here, in the absence of Bim, scar formation continued to increase without impacting the level of CNV. CNV and scar formation are intertwined to varying degrees. The question that arises is whether using an antifibrotic agent is sufficient to inhibit not only fibrosis but also neovascularization. Previous studies have shown that the antifibrotic agent anti-CTGF also inhibits CNV [32]. Thus, while it is likely that treatment to inhibit CNV may be needed in conjunction with antifibrotic therapies for the treatment of nAMD, we believe that an antifibrotic agent may be sufficient to inhibit scar formation by itself. Our data further illustrate the importance of immune cell clearance and the potential use of senolytic compounds to preserve vision. The detailed mechanisms will require further investigation. Accumulating subretinal scars decrease visual acuity and are associated with an incomplete response to treatment in at least 1/3 of nAMD patients receiving anti-VEGF therapy. Thus, gaining a better understanding of how subretinal scars are initiated and continue to form will aid the design of better treatment modalities for these patients.

## 4. Materials and Methods

### 4.1. Animals

We obtained breeder pairs for global Bim +/- mice from Jackson Laboratory (stock number 004525; Bar Harbor, ME, USA), which were then maintained at the University of Wisconsin animal facilities. These mice were genotyped using 5′-CATTCTCGTAAGTCCGAGTCT-3′,5′-GTGCTAACTGAAACCAGATTAG-3′ and 5′-CTCAGTCCATTCATCAACAG-3′ as primers. Conditional Bim mice received from Dr Andreas Strasser and Dr Phillipe Bouillet were screened using the following primers: 5′-AACCAACTGTACCTT GGCTATA-3′ and 5′-GACAAGGTGGACAA TTGCAG-3′. These Bim^Flox/Flox^ mice were healthy [14] and had been previously characterized and utilized by us and others [14,42,43,44,45,46,47]. We crossed Bim^Flox/Flox^ with VE-cadherin-Cre (B6.Cg-Tg(Cdh5-cre)7Mlia/J; Jackson stock number 006137) or Lyz2-Cre (Jackson stock number 004781) mice. The progenies that were Bim^Flox/+^ and expressed VE-cadherin-Cre or Lyz2-Cre were crossed next. For VE-cadherin-Cre, genotyping was carried out with the following primers: 5′-GCGGTCTGGCAGTAAAAACTATC-3′ and 5′-GTGAAACAGCATTGCTGTCACTT-3′. For Lyz2-Cre, we used 5′-CCCAGAAATGCCAGATTACG-3′, 5′-CTTGGGCTGCCA GAATTTCTC-3′, and 5′-TTACAG TCGGCCAGGCTGAC-3′ as primers. We finally generated Bim^Flox/Flox^ mice that also expressed VE-cadherin-Cre (endothelial-cell-targeted mice are called Bim^EC^) or Lyz2-Cre (mononuclear-phagocyte-targeted mice are called Bim^MP^). 

### 4.2. Laser-Induced Choroidal Neovascularization (CNV)

Male and female (3-month-old) mice were anesthetized with ketamine hydrochloride (80 mg/kg) and xylazine (10 mg/kg), and their pupils were dilated with tropicamide (1%) on the day of laser photocoagulation. An OcuLight GL diode laser fitted with a slit lamp delivery system (Iridex, Mountain View, CA, USA) was utilized to locate positions 9, 12, and 3 o’clock on the posterior pole of each eye. Laser photocoagulation (75 µm spot size, 0.1 sec duration, 120 mW) was accomplished with a handheld cover slip to view the retina and facilitate Bruch’s membrane rupture. To assess the choroid–RPE complex neovascularization 5 weeks post laser photocoagulation, the complex was fixed in 4% paraformaldehyde (PFA) and incubated with blocking buffer (20% normal goat serum and 5% fetal calf serum in 1x PBS) for 1 h, then incubated with anti-ICAM-2 (BD Pharmingen; #553326; 1:500 in 1x PBS with 20% normal goat serum and 20% fetal calf serum) at 4 °C overnight. To assess MPs 3, 6, and 14 days following laser photocoagulation, the RPE–choroid complex was stained with anti-F4/80 (eBioscience #14-4801-82; 1:500), anti-Iba1 (Invitrogen # PA5-27436; 1:500), anti-CD11b (BD Pharmingen #557393; 1:500), or anti-CD80 (eBioscience #11-0801-81; 1:500). To assess scar formation, the RPE–choroid complex was stained with anticollagen I (Abcam #ab34710; 1:500). Following staining with the primary antibody, the appropriate secondary antibody (Jackson ImmunoResearch, West Grove, PA, USA 1:500) was added if needed, and a Zeiss microscope (Zeiss, Chester, VA, USA) captured images in digital format. We measured the total stained areas by pixel intensities (in µm^2^) with Image J software last accessed 20 September 2021 (National Institute of Mental Health, Bethesda, MD, USA; http://rsb.info.nih.gov/ij/, accessed on 20 September 2021).

### 4.3. Data Analysis

Statistical differences between two groups were evaluated with the Student’s unpaired t-test (two-tailed). We evaluated statistical differences between more than two groups with one-way ANOVA followed by Tukey’s Multiple Comparison Test using GraphPad Prism 8.0 (GraphPad Software, San Diego, CA, USA). Tukey’s Multiple Comparison Test was performed to determine the significant differences between the means of every possible two groups in all experimental groups of three or more [48]. Mean ± SEM is shown in the figures. A value of *p* < 0.05 was considered significant. 

## 5. Conclusions

In conclusion, our study demonstrates that MP clearance plays an essential role in reducing ongoing scar formation. In our studies, this appeared to be separable from neovascularization. When Bim is lacking globally, in endothelial cells or MPs, immune cells persist, leading to increased scar formation. 

## Data Availability

All the data in support of the findings presented are included here and can be shared by the corresponding author with reasonable requests.
